# Climate Change Influences the Spread of African Swine Fever Virus

**DOI:** 10.3390/vetsci9110606

**Published:** 2022-11-01

**Authors:** Shraddha Tiwari, Thakur Dhakal, Tae-Su Kim, Do-Hun Lee, Gab-Sue Jang, Yeonsu Oh

**Affiliations:** 1 Department of Veterinary Pathology, College of Veterinary Medicine and Institute of Veterinary Science, Kangwon National University, Chuncheon 24341, Korea; 2 Department of Life Science, Yeungnam University, Daegu 38541, Korea; 3 National Institute of Ecology (NIE), Seocheon 33657, Korea

**Keywords:** ASFV, Maxent, wildlife, disease, management

## Abstract

**Simple Summary:**

This study aims to investigate the influence of climate change on the spread of the African swine fever virus (ASFV). ASFV data in wild boar outbreak locations were sampled and investigated using the Maxent model, with WorldClim bioclimatic data as the predictor variables. The future impacts of climate change on ASFV distribution were scoped with Representative Concentration Pathways (RCP) scenarios for 2050 and 2070. The results show that the precipitation of the driest month (Bio14) and annual mean temperature (Bio1) were contributable factors and indicate a higher possibility of spreading ASFV in the future. The Maxent model was best fitted with an area under curve (AUC) value of 0.99. The proposed Maxent model and the results of this study can be potentially applied to predict disease risks associated with climate change and provide guidance for prevention management.

**Abstract:**

Climate change is an inevitable and urgent issue in the current world. African swine fever virus (ASFV) is a re-emerging viral animal disease. This study investigates the quantitative association between climate change and the potential spread of ASFV to a global extent. ASFV in wild boar outbreak locations recorded from 1 January 2019 to 29 July 2022 were sampled and investigated using the ecological distribution tool, the Maxent model, with WorldClim bioclimatic data as the predictor variables. The future impacts of climate change on ASFV distribution based on the model were scoped with Representative Concentration Pathways (RCP 2.6, 4.5, 6.0, and 8.5) scenarios of Coupled Model Intercomparison Project 5 (CMIP5) bioclimatic data for 2050 and 2070. The results show that precipitation of the driest month (Bio14) was the highest contributor, and annual mean temperature (Bio1) was obtained as the highest permutation importance variable on the spread of ASFV. Based on the analyzed scenarios, we found that the future climate is favourable for ASFV disease; only quantitative ratios are different and directly associated with climate change. The current study could be a reference material for wildlife health management, climate change issues, and World Health Organization sustainability goal 13: climate action.

## 1. Introduction 

Monitoring, modelling, and managing (3Ms) are the pillars for understanding specific contexts, challenges, and bottlenecks in epidemiology [1]. African swine fever virus (ASFV) is a re-emerging viral animal disease [2] that is a large double-stranded DNA virus in the *Asfarviridae* family and the causative agent of African swine fever (ASF) with high mortality (100%) rates in pigs [3]. Outbreaks of ASFV have been continuously monitored and analyzed with different modelling approaches and management strategies [4,5]. Since there is no effective vaccine or treatment, vector control and preventive measures are currently the only options for mitigating ASFV outbreaks [6]. Climate change generates more uncertainty and greater susceptibility, and understanding impacts under different climatic scenarios supports setting suitable mitigation actions [7].

The impact of climate change on animal and human health is a highly topical concern, with extensive debates and speculations frequently forecasting the worst [8,9,10]. The World Health Organization (WHO) has concluded that climatic changes have occurred since the mid-1970s [11], which is the shifting of climate patterns particularly caused by greenhouse gases emitted from the natural system as forest fires, earthquakes and volcanoes, and human activities [12,13]. Multiple organizations and institutions have collaborated to provide independent and global monitoring mechanisms to track climate change issues [14,15]. Researchers have contributed different strategies and models for adopting and mitigating climate change [16]. 

Climate change threatens biodiversity, mainly habitat loss, natural disasters, human-wildlife conflict, and species extinction [17,18,19] and escalates the risk of infectious disease outbreaks [20,21]. The spread of diseases has challenged ecologists to understand the host-parasite interactions and driving factors [22]. Previous studies reported that human and animal pathogenic viruses such as West Nile virus (WNV), herpes simplex (HSV), rabies, Chikungunya virus (CHIKV), novel coronavirus infections (COVID-19 or SARS-CoV-2), Rift Valley fever virus (RVFV), African swine fever virus (ASFV), and Bluetongue virus (BTV) have emerged as a result of climate-induced changes in vegetation, human activities [23,24,25], and a variety of contributory determinants [26,27,28]. However, significant research and models for analyzing the relationship of climate change to disease vector populations and epidemiology still require more research for proper control [29].

Long-term studies of climate factors may reveal the association between ASF and climate change [7]. Similarly, it is imperative to know the distribution pattern but hard to determine for environmental management. Various modelling approaches have been used on theoretical ecology, on climate change and conservation policy impact, and for planning purposes [30]. In recent years, the maximum entropy model (Maxent) has been the most popular and broadly used algorithm for analyzing distribution with presence-only data [30,31]. The spatial distribution of pests and some diseases using the Maxent model has been extensively studied [32,33,34,35,36,37,38,39,40,41]. However, the climate impact on wildlife disease ASFV spread in the global scenario is still limited. 

Pathogenic diseases can be exacerbated by climate change [42], and bioclimatic indices are used to examine the risk of suffering from heat stress [43]. We hypothesized that multiple bioclimatic scenarios could illustrate the possible risk of ASFV. This study examines the current and future distribution of ASFV globally using the Maxent model [44] and ASFV in wild boar outbreaks locations recorded from 1 January 2019 to 29 July 2022 with 19 bioclimatic predictors. The predictive ASFV risk for 2050 and 2070 in different greenhouse gas concentration trajectories and representative concentration pathway (RCP) [45] scenarios have been reported. These findings will aid in demonstrating the spread of ASFV, identifying hidden high-risk areas, and improving the efficacy of wildlife disease management. 

## 2. Materials and Methods 

### 2.1. Data

#### 2.1.1. Presence Data 

ASF was first detected in East Africa in Kenya in the early 1900s, later spread to Europe in the late 1950s and started to spread in Asian countries in 2018 [2,46]. Wild boars freely move in the forest and are more susceptible to ASFV than domestic pigs [47]. Therefore, we sampled the global ASFV wild boar outbreaks data from 1 January 2019 to 29 July 2022 mined from the Food and Agriculture Organization (FAO) of the United Nations data portal https://empres-i.apps.fao.org/epidemiology (accessed on 30 July 2022) [48] for Maxent analysis. The portal is the FAO’s updated global health intelligence and early warning platform to improve forecasting and allow countries to track the spread of the virus and the risk of new outbreaks in different livestock species. Initially, we obtained a total of 17,828 coordinates, and after removing the repeated outbreak locations, 16,186 points were analyzed in this study. 

#### 2.1.2. Climatic Variables 

Nineteen historical bioclimatic feature raster data, most commonly used over a long-time frame (from 1970 to 2000, considered as current data released in January 2020), were extracted from the WorldClim dataset [49] using the ‘getData’ function of the ‘Raster’ library in Rstuio [50,51]. Similarly, future bio variables of 2050 and 2070 on different RCPs (RCP 2.6, 4.5, 6.0, and 8.5) [45] were downloaded from the Coupled Model Intercomparison Project Phase 5 (CMIP5) [52] application programming interface (API) in Rstuio [50,51]. The corresponding current 19 bioclimatic features [49] (climatic data) of each outbreak location were extracted, and collinearity problems [53] were checked with the threshold of correlation coefficient 0.8. After checking the collinearity test using a variable infection factor (VIF), ten features (**Bio**1, **Bio**2, **Bio**3, **Bio**7, **Bio**8, **Bio**9, **Bio**10, **Bio**12, **Bio**14, and **Bio**15) were selected and used for further analysis (bold text in Table 1). Bio5, Bio6, and Bio7 were the highest VIF coefficients (Inf) variables and the lowest of Bio8 (6.17).

### 2.2. Modelling Approach and Evaluation

Maxent is the density estimation, where probability distribution π over a set of data X of the survey area is examined from the outbreak locations and environmental variables based on the Bayesian rule (Equation (1)). The presence data in set *X* is considered as 1 and 0 for absence as response variable *Y*, and the distribution *π* (*X*) is the conditional probability *P*(*X*|*Y* = 1) [54].

(1) $$P\left(Y=1|X\right)=\frac{P\left(X|Y=1\right)P\left(Y=1\right)}{P\left(X\right)}=\pi \left(X\right)P\left(Y=1\right)\left|X\right|$$

In this study, we used the Maxent model suggested by Maxent [44] using the ‘dismo’ library in Rstudio [51]. The presence data were grouped randomly for 75% training (12,949 points) and 25% testing (3237 points), and the model was trained with the training dataset for 5000 replications. Maxent contracts presence against pseudo-absence background data that need to be greater than 10,000 points for higher samples [55]. Therefore, we used 10,000 random background locations to evaluate the model with a testing data set. Current bioclimatic variables have been used as environmental predictors for training and testing the model.

The model’s performance was evaluated using the area under the curve (AUC) [44], Cohen’s Kappa [56], and True skill statistics (TSS) [57] values in measuring the distribution of ASFV outbreak locations. AUC values range between 0 and 1; 0.9–1.0 was considered excellent, 0.8–0.9 good, 0.7–0.8 fair, and 0.7 poor [58]. Cohen’s Kappa values range between −1 and +1, whereas 0.80–1.0 suggests excellent, 0.60–0.80 as substantial, 0.40–0.60 as moderate, 0.20–0.40 as fair, 0.01–0.20 as non to slight, and ≤0 as no agreement [59]. TSS values range from 1 to +1, with +1 indicating perfect agreement and a value less than or equal to zero, indicating the performance is not superior to random [57]. 

**Figure 1:**
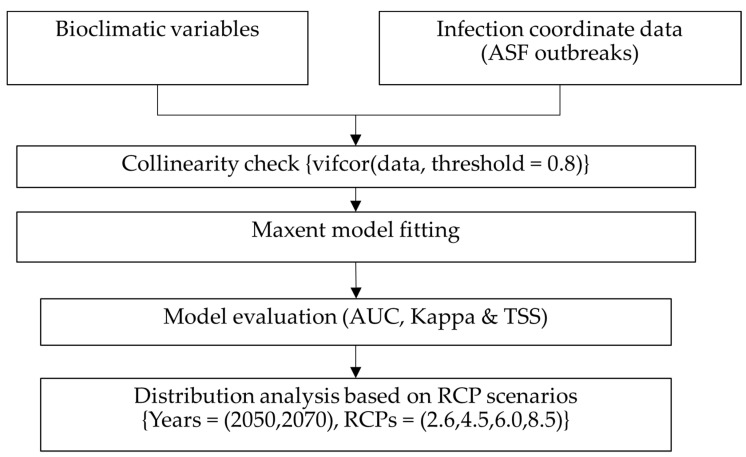
Study flow.

The percentage contribution and permutation importance [60] of the ASFV suitability have been evaluated from the optimal fitted model. The model was further used to predict the possible distribution of ASFV worldwide under different RCP scenarios. The average complementary log-log (cloglog) output values [61] for each future RCP and current distribution maps were compared, and possible influences due to climate change were reported. Finally, since entropy provides average information about the event, the higher the entropy value, the greater the suitability and higher probability of diffusion [44,62]; we concluded the influences of climatic change on spreading ASFV. The detailed study flow is illustrated in Figure 1.

## 3. Results and Analysis 

The Maxent model was fitted with ASFV outbreak point data and ten climatic predictors conducted after the collinearity test. Based on the performance measures that we considered in the study, we could say the examined model performed excellent prediction through AUC (0.99) and TSS (0.77) and substantial agreement on Kappa (0.60) tests. Two variable contribution measures, relative percentage contribution and permutation importance [60] with ten bioclimatic features, were investigated from the optimal model (Table 2). Bio14 highly contributed (49.3%) to the spread of ASFV, followed by Bio1 (34%), Bio9 (5.5%), Bio12 (3.1%), and, the lowest, Bio8 (0.2%). Similarly, the highest permutation was obtained for Bio1 (29.4%), followed by Bio2 (23.8%), Bio14 (17%), and a negligible (0%) effect by Bio8. 

**Figure 2:**
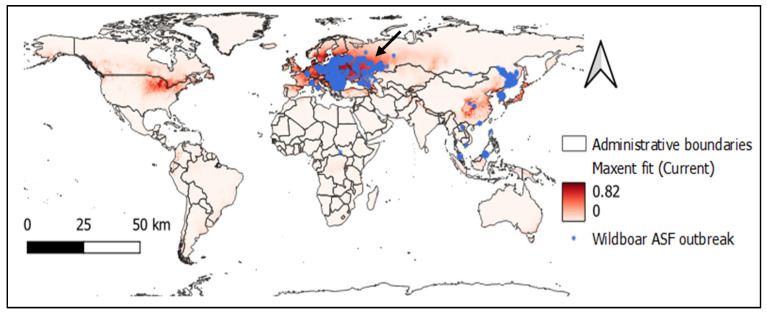
Maxent predicted map with current bioclimatic features and wild boar ASF outbreak locations (black arrow points to Europe as the recent hotspot of ASFV).

Based on the observed model, the potential global distribution of ASFV with presence occurrence data and current bioclimatic variables can be seen in Figure 2. The Maxent output entropy values with current climatic conditions in the world ranged between 0 and 0.82 (mean 0.06), and the potential distribution area is in the northern hemisphere. 

**Figure 3:**
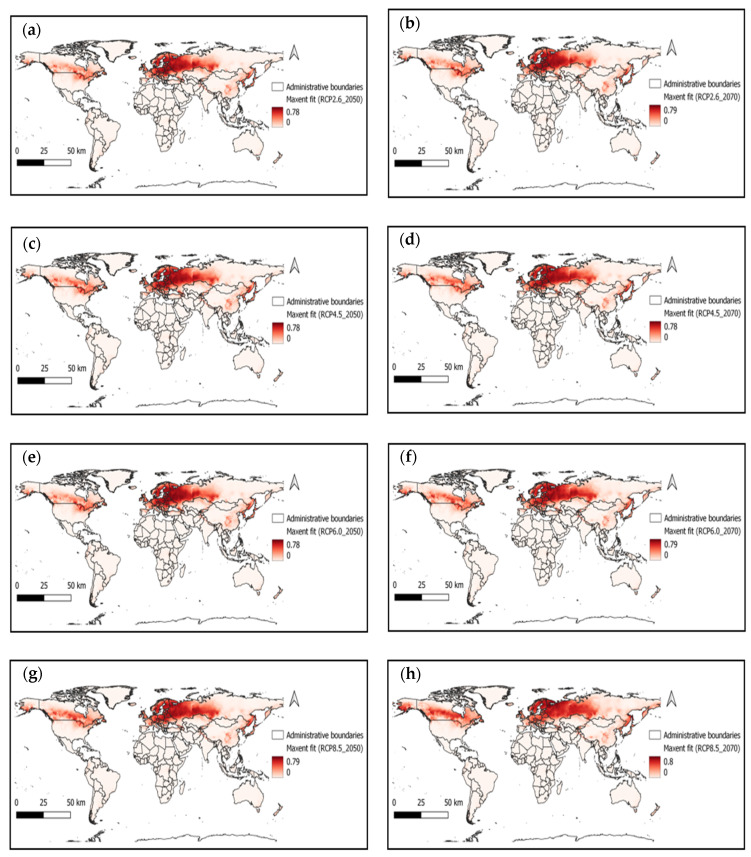
ASFV suitability map with different (**a**) RCP 2.6_2050, (**b**) RCP 2.6_2070, (**c**) RCP 4.5_ 2050, (**d**) RCP 4.5_ 2070, (**e**) RCP 6.0 _ 2050, (**f**) RCP 6.0 _2070, (**g**) RCP 8.5_ 2050, and (**h**) RCP 8.5_ 2070 scenarios.

We have examined the impact of future climate conditions on the suitability of ASFV from the above optimal Maxent model. The probable impact maps with different scenarios for the years 2050 and 2070 were captured in Figure 3. For each scenario (RCP 2.6_2050 (Figure 3a), RCP 2.6_2070 (Figure 3b), RCP 4.5_ 2050 (Figure 3c), RCP 4.5_ 2070 (Figure 3d), RCP 6.0 _ 2050 (Figure 3e), RCP 6.0 _2070 (Figure 3f), RCP 8.5_ 2050 (Figure 3g), and RCP 8.5_ 2070 (Figure 3h)), the Maxent entropy value ranges slightly decreased compared to the current scenario but increased the suitability zones.

**Figure 4:**
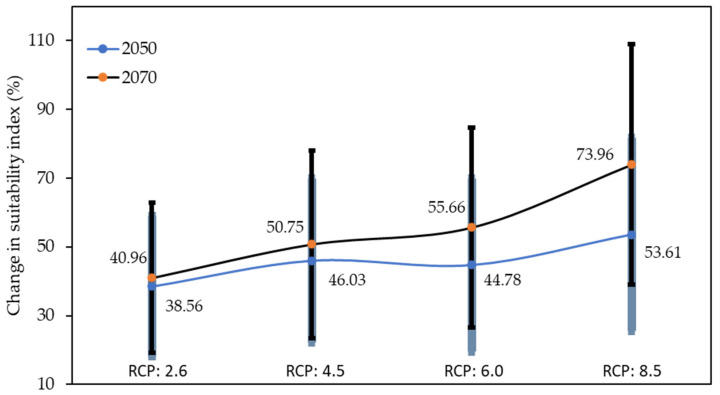
Average change (percent) of ASFV suitability index and standard deviation (error bars) in different scenarios from current year to 2050 and 2070.

We further examine the quantitative influence of climate changes on the suitability of ASFV based on the average Maxent entropy value and error as their standard deviation (Figure 4). The average suitability index in 2050 for RCPs 2.6, 4.5, 6.0, and 8.0 increased by 38.56, 46.03, 44.78, and 53.61 percent over the current index, respectively. The highest risk of ASFV in 2050 can be seen in RCP 8.5, followed by RCPs 4.5, 6.0, and 2.6. Similarly, the highest risk of ASFV in 2070 compared with the current index can be seen in RCP 8.5 (73.96%) followed by RCPs 6.0 (55.66%), 4.5 (50.75%), and 2.6 (40.96%). Overall, we can see that risk of ASFV is higher than in the current climatic conditions, and the RCP 6.0 (44.78%) scenario is a relatively lower risk than RCP 4.5 (46.03%) in 2050. With these changes in bioclimatic values under different scenarios, ASFV suitability indices have been changed, which infers evidence of climate change’s impact on the spread of ASFV.

## 4. Discussion 

Climate change is today’s central concern that must be mitigated for a sustainable ecosystem [63]. Climate change increases cross-species viral sharing [64]. ASF is a socio-economic burden on biodiversity and food security. Swine industries worldwide have the biggest threat from ASF [65]. Analyzing this threat linking climate change and its potential distribution could be a supportive reference for wildlife and pig industry management. Identifying possible factors and modelling approaches could support the control of epidemic spread. Suitability modelling is a powerful tool for analyzing the potential distribution that can help to design ecology and epidemic management policies [1,44]. Ecological niche models have been broadly used for risk analysis in epidemiology [66]. 

This study analyses the possible impact of climate changes on the suitability of ASFV through the commonly used ecological distribution model, Maxent [30]. Maxent deals with presence-only data [54]; 16,186 locations of ASFV in wild boar outbreaks were analyzed with current [49] and predicted CMIP5 [52] RCP scenarios’ bioclimatic data. Initially, we checked the collinearity problem with the current 19 bioclimatic variables and presence data and obtained ten eligible bio variables for the Maxent model. The model was fitted with outbreak data and ten bioclimatic variables and predicted with an optimal model for current and future climatic data. The observed model had AUC (0.99), TSS (0.77), and Kappa (0.60), which confirmed that Maxent could effectively estimate the probability of risk of ASF. 

Climatic and environmental factors influence ASFV. The number of ASFV infections in pigs decrease in summer temperatures below 16 °C, and outbreaks are higher in locations above −1 °C [67]. Our study investigated the impact and influenceable climatic factors of ASFV spread and probable future scenarios for 2050 and 2070 with different RCP conditions. We found that the precipitation of the driest month (Bio 14) and annual mean temperature (Bio 1) were the primary contributors to ASFV distribution. For each future scenario, average suitability indexes were found to be higher than the current climatic conditions (see Figure 3 and Figure 4).

The future climatic RCP scenarios have different trends. RCP 2.6 is the straight pathway where carbon emission will decline by 2020 and reach zero by 2100; RCP 4.5 is moderate: emission will peak about 2040 and then decline. Similarly, the emission rate is high, peaking around 2080 in RCP 6.0, declining, and continuously increasing through the 21st century in 8.5 [68]. Bioclimatic factors are climate change-induced variables derived from temperature and precipitation and are extensively used for ecological modelling [49]. Observing the current occurrence locations, future distributions can be predicted using data-driven models [36,54]. Based on the available two-period bioclimatic data of different RCP pathways by 2050 and 2070 and data-driven maxent estimations, a higher risk of ASFV was observed with climate change. 

This study illustrates climate change in the spread of ASFV with bioclimatic scenarios and the Maxent model; however, there are significant limitations. The impact of climate change on ASFV is complex, and outbreak locations could be driven by multiple factors that make general predictions impossible. The spread of disease is influenced by various factors such as movement behaviour, the habitat and population density of reservoir and novel host populations [69], climate and land use changes [70], and anthropogenic factors [71], but the current study only examined the climatic covariates. Detailed studies considering disease-driving factors, including domestic pig cases, would present better output. In the future, more variables with heterogeneous effects on ASFV distribution could be analyzed. The economic value of one health approach impacting disease spread [72] also needs research to control climate change and disease as ASFV diffusion. The current study used the Maxent model to investigate the effect of climatic change; comparison, validation with ensemble models [73], and hybrid data-driven-mechanistic models [1,74] could be the better-represented model that warrants further investigation. The changes in bioclimatic variable data influenced entropy values, and the study advocated that climate change influences the spread of ASFV: further investigation and validation must be undertaken.

## 5. Conclusions

In this study, we analyzed the ASFV in wild boar outbreak locations to fit the Maxent model with ten bioclimatic variables after removing nine variables that created the collinearity problems and examined the possible impact of climatic variables for 2050 and 2070 with multiple RCP scenarios. The results show climatic changes are influencing the spread of ASFV, and the future climate is favourable for ASFV spread: only quantitative ratios are different. Based on the observed model with climatic variables, the spread of wild disease ASFV is directly associated with climate changes. There is no vaccine to eradicate ASF, so improving biosafety measures is currently the best option to prevent ASFV [75,76]. We have emphasized the importance of ASFV control management in conjunction with climate change. Mitigation of climatic change supporting the WHO sustainability goal 13 (Climate action) [77] is to be strictly implemented to control the risk of ASFV. WHO and OIE have recommended operational strategies for maintaining essential human and animal health services that must be assessed and reported [78]. The implication of distribution model predictions can be a supportive tool to deal with the future distribution of wildlife diseases like ASFV. The current study could be a reference to global health concerns.

## Figures and Tables

**Table 1:** The bioclimatic features, their codes, VIF values and units.

Bioclimatic Variables *	Code	VIF Value	Unit
**Annual Mean Temperature**	**Bio** **1**	309.80	°C
**Mean Diurnal Range (Mean of monthly (max temp–min temp))**	**Bio2**	72.45	°C
**Isothermality (Bio2/Bio7) (×100)**	**Bio3**	17.20	%
Temperature Seasonality (standard deviation ×100)	Bio4	1435.91	°C
Max Temperature of Warmest Month	Bio5	Inf	°C
Min Temperature of Coldest Month	Bio6	Inf	°C
**Temperature Annual Range (Bio5-BIO6)**	**Bio7**	Inf	°C
**Mean Temperature of Wettest Quarter**	**Bio8**	6.17	°C
**Mean Temperature of Driest Quarter**	**Bio9**	8.11	°C
**Mean Temperature of Warmest Quarter**	**Bio10**	937.86	°C
Mean Temperature of Coldest Quarter	Bio11	1471.54	°C
**Annual Precipitation**	**Bio12**	294.04	mm
Precipitation of Wettest Month	Bio13	211.56	mm
**Precipitation of Driest Month**	**Bio14**	65.35	mm
**Precipitation Seasonality (Coefficient of Variation)**	**Bio15**	77.61	%
Precipitation of Wettest Quarter	Bio16	801.18	mm
Precipitation of Driest Quarter	Bio17	104.71	mm
Precipitation of Warmest Quarter	Bio18	334.52	mm
Precipitation of Coldest Quarter	Bio19	29.94	mm

* Variables after collinear test at correlation threshold value 0.8 are in bold text.

**Table 2:** Variable contribution for ASFV distribution.

Variable	Percent Contribution	Permutation Importance
Bio14	49.3	17
Bio1	34	29.4
Bio9	5.5	0.7
Bio12	3.1	7.8
Bio3	2.8	3.6
Bio10	2.2	7.1
Bio2	1.9	23.8
Bio15	0.7	5
Bio7	0.5	5.4
Bio8	0.2	0

## Data Availability

The data presented in this study are available from the corresponding author upon request.
